# National Surveillance of Tetracycline, Erythromycin, and Clindamycin Resistance in Invasive *Streptococcus pyogenes*: A Retrospective Study of the Situation in Spain, 2007–2020

**DOI:** 10.3390/antibiotics12010099

**Published:** 2023-01-06

**Authors:** Pilar Villalón, Marta Bárcena, María José Medina-Pascual, Noelia Garrido, Silvia Pino-Rosa, Gema Carrasco, Sylvia Valdezate

**Affiliations:** Laboratorio de Referencia e Investigación en Taxonomía, Centro Nacional de Microbiología, Instituto de Salud Carlos III, Majadahonda, 28220 Madrid, Spain

**Keywords:** *Streptococcus pyogenes*, genotype, phenotype, antimicrobial resistance, tetracycline, erythromycin, clindamycin, *emm* type, clone

## Abstract

Background: This work reports on antimicrobial resistance data for invasive *Streptococcus pyogenes* in Spain, collected by the ‘Surveillance Program for Invasive Group A *Streptococcus*’, in 2007–2020. Methods: *emm* typing was determined by sequencing. Susceptibility to penicillin, tetracycline, erythromycin, and clindamycin was det*erm*ined via the E-test. *tet*M, *tet*O, *msr*D, *mef*A, *erm*B, *erm*TR, and *erm*T were sought by PCR. Macrolide-resistant phenotypes (M, cMLSB, and iMLSB) were detected using the erythromycin–clindamycin double-disk test. Resistant clones were identified via their *emm* type, multilocus sequence type (ST), resistance genotype, and macrolide resistance phenotype. Results: Penicillin susceptibility was universal. Tetracycline resistance was recorded for 237/1983 isolates (12.0%) (152 carried only *tet*M, 48 carried only *tet*O, and 33 carried both). Erythromycin resistance was detected in 172/1983 isolates (8.7%); *erm*B was present in 83, *mef*A in 58, *msr*D in 51, *erm*TR in 46, and *erm*T in 36. Clindamycin resistance (methylase-mediated) was present in 78/1983 isolates (3.9%). Eight main resistant clones were identified: two that were tetracycline-resistant only (*emm*22/ST46/*tet*M and *emm*77/ST63/*tet*O), three that were erythromycin-resistant only (*emm*4/ST39/*mef*A-*msr*D/M, *emm*12/ST36/*mef*A-*msr*D/M, and *emm*28/ST52/*erm*B/cMLSB), and three that were tetracycline–erythromycin co-resistant (*emm*11/ST403/*tet*M-*erm*B/cMLSB, *emm*77/ST63/*tet*O-*erm*TR/iMLSB, and *emm*77/ST63/*tet*M-*tet*O-*erm*TR/iMLSB). Conclusions: Tetracycline, erythromycin, and clindamycin resistance rates declined between 2007 and 2020. Temporal variations in the proportion of resistant clones det*erm*ined the change in resistance rates.

## 1. Introduction

*Streptococcus pyogenes*, also called group A *Streptococcus*, causes illnesses of different severities. Invasive infections are less common than superficial infections but are associated with higher mortality [1,2]. A large number of virulence factors contribute to the pathogenicity of *S. pyogenes*, including the presence of the surface M protein and a set of exotoxins, the members of which act as superantigens [2,3]. The microbiological typing of strains is based on the *emm* gene (which codes for the M protein), which determines the *emm* type (formerly called the serotype) [3]. Superantigens can trigger an uncontrolled immune response known as a cytokine storm, which plays an essential role in the onset of STSS and necrotizing fasciitis, some of the most severe infections [2,3].

Penicillins/beta-lactams are the first-line treatments for *S. pyogenes* invasive infections; clindamycin in combination with penicillin is recommended for the most severe infections due to clindamycin’s antitoxic activity. Macrolides, vancomycin, and linezolid can replace beta-lactams in intolerant patients. Macrolides are also commonly used in superficial infections, such as pharyngitis [4,5]. Although tetracyclines are not used to treat *S. pyogenes* infections, resistance knowledge is epidemiologically important. Macrolide and tetracycline resistance genes are basically acquired by horizontal transfer [6,7,8]. Temporal variations in antimicrobial resistance genes (ARGs) are related to temporal variations in *emm* types carrying these genes [9,10,11,12,13]. The study of acquired ARGs is, therefore, essential in defining the circulating clones.

*S. pyogenes* remains susceptible to penicillin [2]. The macrolide resistance mechanisms most commonly encountered involve the MefA–MsrD active efflux pump and Erm methylases [2,14,15]. *mef*A and *msr*D are part of the same operon and are involved in the expulsion of 14- and 15-membered ring macrolides; they are responsible for the macrolide-resistant M phenotype [15]. The *erm* genes code for proteins that methylate 23S rRNA, modifying the binding site of macrolides, lincosamides, and streptogramin B (MLSB). This results in cross-resistance among these antibiotics and the expression of the macrolide-resistant MLSB phenotype, which can be constitutively expressed (cMLSB) or inducibly expressed in the presence of erythromycin (iMLSB) [14,15]. The methylase genes possessed by *S. pyogenes* include the chromosomal *erm*B [14,15] and *erm*TR [15,16], in addition to the plasmidic *erm*T [15,17]. Tetracycline resistance is effectively due to ribosomal protection proteins encoded by *tet*M and *tet*O [2,15]. Tetracycline and macrolide co-resistance is usual in *S. pyogenes*, a consequence of the presence of genetic elements—i.e., phages, transposons, plasmids, conjugative integrative elements, etc.—that carry resistance determinants for both groups of antibiotics [6,7,8,9].

The surveillance of *S. pyogenes* is important; only then can the characteristics of the circulating strains be known, changes in trends detected, and early control as well as preventive measures put in place. The ‘Surveillance Program for Invasive Group A *Streptococcus*’ (SPIGAS) has been implemented by the National Centre for Microbiology (NCM) [18] since 1994, and its main objective is to obtain microbiological information on invasive strains circulating in Spain [19] through phenotypic, genotypic, and/or genomic research. All Spanish hospitals and public health laboratories can voluntarily take part in this free of charge program.

In Spain, in the period of 2007–2019, the most prevalent invasive *emm* types—*emm*1, *emm*89, and *emm*3—were basically susceptible to antimicrobials [19]. Here, we analyze the less frequently resistant *emm* types, the ARGs, resistance rates, and trends; we also compare our results with those of previous studies.

Using the genotype and phenotype information for the resistant *emm* types detected by SPIGAS, the aim of the present work is to provide better knowledge on the tetracycline, erythromycin, and clindamycin resistance shown by invasive *S. pyogenes* in Spain in the period of 2007–2020.

## 2. Results

### 2.1. Clinical and Epidemiological Data for Resistant S. pyogenes Isolates

In the period 2007–2020, (*n* = 1983) *S. pyogenes* isolates that caused invasive illness were analyzed by SPIGAS. A total of 315 (15.9%) were resistant to tetracycline, erythromycin, and/or clindamycin. These resistant isolates were detected in 13/17 of Spain’s autonomous regions (involving 29/50 of the country’s provinces). The affected age range was 0–99 years, with a median of 47 years; patients ≥ 75 years of age (*n* = 61, 19.4%) made up the most affected age group. A total of 168 (53.3%) patients were male, and 139 were (44.1%) female; for 8 patients (2.5%) no gender information was available. The most commonly examined samples were blood (136 samples, 43.2%) and wound exudate (106, 33.7%). Clinical manifestations included sepsis (84, 26.7%), cellulitis (70, 22.2%), wound infections (33, 10.5%), ulcers (21, 6.7%), arthritis (20, 6.3%), scarlet fever (18, 5.7%), pneumonia (16, 5.1%), necrotizing fasciitis (11, 3.5%), abscesses (11, 3.5%), and others (31, 9.8%).

### 2.2. emm Types Resistant to Tetracycline, Erythromycin, and Clindamycin

**Figure 1:**
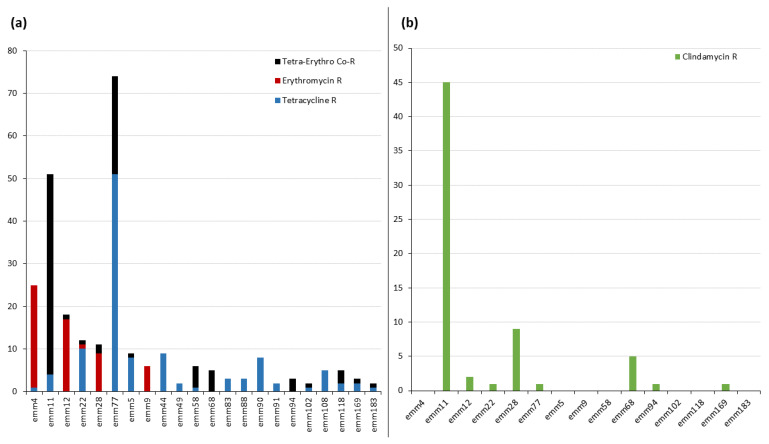
Antimicrobial-resistant *Streptococcus pyogenes emm* types. (**a**) *emm* types resistant to tetracycline and erythromycin. (**b**) Clindamycin resistance in erythromycin-resistant *emm* types. Vertical axis = number of isolates. R, resistance.

All isolates were susceptible to penicillin; MIC_50_ and MIC_90_ values of 0.016 and 0.023 mg/L were recorded, respectively. Table 1 shows the data for susceptibility to tetracycline, erythromycin, and clindamycin for the six majority *emm* types (>10 resistant isolates) significantly associated (*p* ≤ 0.05) with some kind of resistance. Tetracycline resistance was observed in 237 isolates (12.0%). *emm11, emm22*, and *emm77* were the majority *emm* types associated with resistance to this antibiotic; significantly associated minority *emm* types (≤ 10 resistant isolates) included *emm5, emm44, emm49, emm58, emm68, emm83, emm88, emm90, emm91, emm94, emm102, emm108, emm118, emm169,* and *emm183*. Erythromycin resistance was detected in 172 isolates (8.7%), mostly associated with *emm4, emm11, emm12,* and *emm77* (majority types), but also with *emm9, emm58, emm68, emm94*, and *emm118* (minority types). Clindamycin resistance was recorded for 78 isolates (3.9%); this was mostly associated with *emm11* and *emm28* (majority), but also with *emm68* (minority). Finally, co-resistance to tetracycline and erythromycin (4.9% of isolates) was associated mostly with *emm11* and *emm77* (majority), but also with *emm58* and *emm68* (minority). Figure 1 shows the *emm* type distribution of isolates resistant to tetracycline, erythromycin, and clindamycin.

### 2.3. Tetracycline and Erythromycin Resistance: Genotype Analysis

Among the tetracycline-resistant population, the *tet*M gene was detected in 185 (78.1%) isolates, while *tet*O was detected in 81 (34.2%). Among the erythromycin-resistant population, the *erm*B gene was detected in 83 isolates (48.3%), *mef*A in 58 (33.7%), *msr*D in 51 (29.7%), *erm*TR in 46 (26.7%), and *erm*T in 36 (20.9%). An association was seen between the *mef*A and *msr*D genes (*p* < 0.00001; relative risk, RR = 17.3), between *erm*B and *tet*M (*p* = 0.0002; RR = 2.2), and between *erm*TR and *tet*O (*p* = 0.0002; RR = 2.9). More than 20 different resistant genotypes were detected among the total 315 examined isolates. Table 1 shows the *emm* types and the most common genotypes associated with tetracycline, erythromycin, and clindamycin resistance. A total of 126 erythromycin-resistant isolates (73.2%) carried the genes for just one macrolide resistance mechanism (Table 1), while the rest (26.8%) carried the genes for two or more. Among the latter, *erm*T was the most common gene detected (possessed by 76% of isolates).

### 2.4. Macrolide and Lincosamide Resistance: Phenotype Analysis

**Figure 2:**
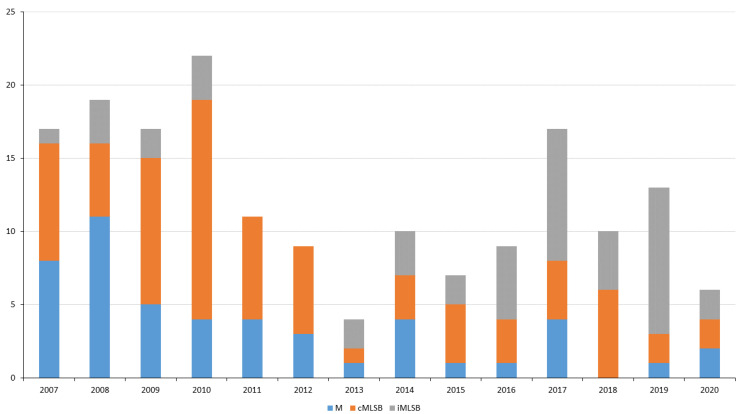
*Streptococcus pyogenes* macrolide-resistant phenotypes. Distribution by year of isolates expressing the M, cMLSB, and iMLSB phenotypes. Vertical axis = number of isolates.

The cMLSB phenotype was detected in 76 (44.1%) isolates resistant to erythromycin, of which 96.1% carried the *erm*B gene. The M phenotype was identified in 49 (28.5%) isolates; 95.9% of these carried *mef*A–*msr*D. The iMLSB phenotype was present in 46 (26.7%) isolates, most of which (82.6%) possessed *erm*TR. No erythromycin-susceptible but clindamycin-resistant isolates were detected. Figure 2 shows the distribution of macrolide-resistant phenotypes over the study period.

### 2.5. Changes in Resistance in 2007–2020

**Figure 3:**
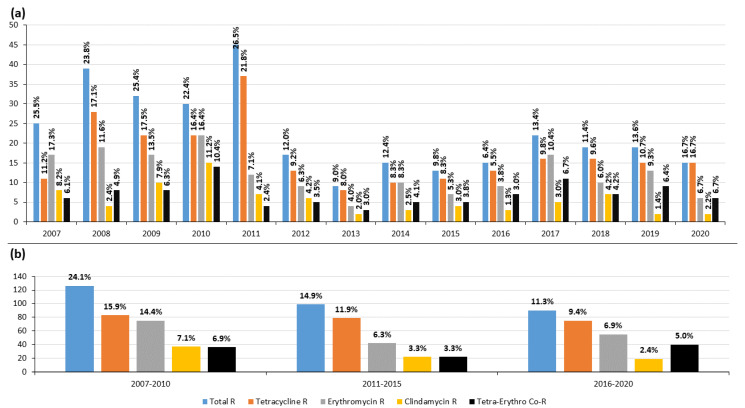
Change over time in antimicrobial resistance in *Streptococcus pyogenes*. Rates of tetracycline, erythromycin, and clindamycin resistance distributed (**a**) yearly and (**b**) by 4–5-year periods. R, resistance. Vertical axis = number of isolates. Total R represents the sum of the resistant isolates.

**Figure 4:**
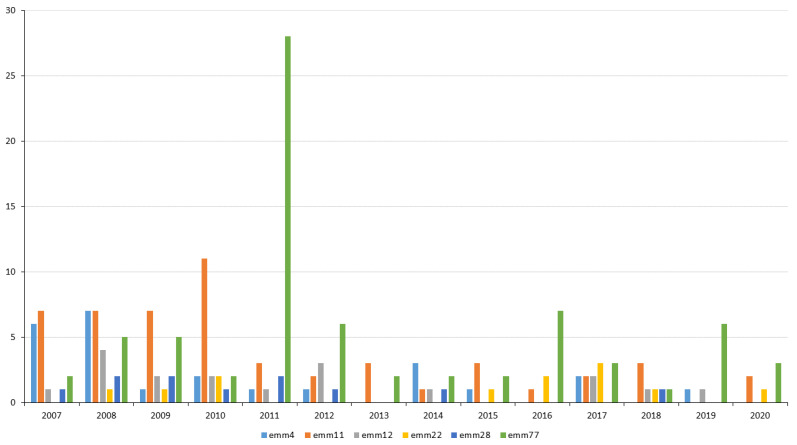
Annual distribution of majority resistant *Streptococcus pyogenes emm* types. Data are for the *emm*4, *emm*11, *emm*12, *emm*22, *emm*28, and *emm*77 resistant isolates. Vertical axis = number of isolates.

Figure 3 shows the changes in antimicrobial resistance from 2007–2020 by year and by 4–5-year periods. In 2007–2010, the resistance rates to tetracycline, erythromycin, and clindamycin were 15.9%, 14.4%, and 7.1%, respectively. In the same order, these rates were 11.9%, 6.3%, and 3.3% in 2011–2015, and 9.4%, 6.9%, and 2.4% in 2016–2020. Figure 4 shows the distribution of resistant *emm* types over time. *emm*4 was more prevalent in 2007–2008, *emm*11 in 2007–2010, *emm*12 in 2008, and *emm*28 in 2007–2012. *emm*22 was the least prevalent majority *emm* type; indeed, it was not detected in 2007, 2011–2014, and 2019. *emm*77 was persistently detected, although it showed annual fluctuations in prevalence, with a remarkably large number of isolates detected in 2011.

### 2.6. Resistant Clones

Table 2 shows the characteristics of the eight most resistant clones. The tetracycline-resistant-only clones were *emm*22/ST46/*tet*M and *emm*77/ST63/*tet*O. Erythromycin-only resistance was recorded for the *emm*4/ST39/*mef*A-*msr*D/M, *emm*12/ST36/*mef*A-*msr*D/M, and *emm*28/ST52/*erm*B/cMLSB clones. The main tetracycline–erythromycin co-resistant clones were *emm*11/ST403/*tet*M-*erm*B/cMLSB, *emm*77/ST63/*tet*O-*erm*TR/iMLSB, and *emm*77/ST63/*tet*M-*tet*O-*erm*TR/iMLSB.

## 3. Discussion

Antimicrobial resistance is a major public health concern and, therefore, is a focus of SPIGAS [18]. Actions must be taken to deal with this problem, such as international and national policies for the better management of antimicrobials, which are being implemented in many countries [21,22]. However, deep knowledge about resistance, its causes, and trends is also necessary. This work tackles these aspects.

The only noteworthy epidemiological difference between the resistant *S. pyogenes* isolates examined in the present work and the susceptible/resistant isolates examined in a previous study [19] was patient age. Infections caused by the present resistant isolates were more common in people ≥ 75 years of age, whereas those caused by the susceptible/resistant isolates of the latter work were most common in 0–4 year olds. This difference may be explained by the fact that the two most representative resistant *emm* types detected in the present work were *emm*11 and *emm*77 (Table 1 and Figure 1), both of which were associated (*p* ≤ 0.05) with infections in elderly people in the 2021 study [19].

Penicillin susceptibility was universal. However, complacency is not recommendable since a few cases of diminished penicillin susceptibility have been described by other authors [23].

Macrolides are the treatment of choice for severe infections in beta-lactam-intolerant patients. Previous studies have shown that erythromycin resistance in *S. pyogenes* has declined in Spain since the 1990s, and that the M phenotype has gradually been replaced by the MLSB phenotype [10,11,12,13,24]. The replacement of phenotypes is mainly explained by changes in the prevalence of *emm*4, *emm*11, and *emm*77—which are associated with the M, cMLSB, and iMLSB phenotypes, respectively (Table 2) [10,11,12,13,24]. The present work searched for the erythromycin resistance genes that have been described for *S. pyogenes* [2,14,15]. Among these, the most prevalent was *erm*B, followed by *mef*A (linked to *msr*D in 51/58 isolates), *erm*TR, and finally *erm*T. The phenotyping and genotyping results agreed (see Table 1) [14,25]. The *erm*B genotype was associated with the strongest macrolide resistance (MICs > 256 mg/L) and nearly always with the cMLSB phenotype, which shows constitutive resistance to erythromycin and clindamycin. The *mef*A–*msr*D genotype showed the least resistance (MICs 16–32 mg/L) and was associated with the M phenotype (i.e., erythromycin-resistant and clindamycin-susceptible), as was also the case for the seven isolates that only carried *mef*A. The *erm*TR genotype basically corresponded to the iMLSB phenotype, which exhibits strong resistance to erythromycin (although less strong than the *erm*B genotype), as well as inducible resistance to clindamycin. In agreement with that which has been previously reported [17], the *erm*T genotype was only detected in one isolate (which expressed the iMLSB phenotype [Table 1]). However, the *erm*T gene was mainly detected in combination with other macrolide resistance genes, especially with the *mef*A–*msr*D–*erm*T, *erm*B–*erm*T, and *erm*TR–*erm*T genotypes, in which its presence did not alter the expression of the M, cMLSB, and iMLSB phenotypes, respectively. How erythromycin resistance is influenced by the presence of *erm*T cannot be concluded from the results of this study, although *erm*T was much more prevalent than expected according to previous work [17]; it is recommended that its presence always be investigated.

Clindamycin in combination with penicillin is of prime importance in the treatment of the most severe *S. pyogenes* infections. Methylase-mediated clindamycin resistance was associated only with the MLSB phenotype [14,15]. Other less common resistance mechanisms involving, e.g., lincosamide nucleotidyltransferases [14,15] were not sought out given the absence of phenotypically compatible isolates.

Tetracycline resistance is of great concern since the *tet* genes are carried on mobile genetic elements (MGEs) that promote their horizontal transmission [6,7,8]. The present results indicate a declining trend in tetracycline resistance over the years, but other recent studies have reported tetracycline resistance rates ranging from 6.8% [12] to 60.6% [9]. Tetracycline resistance (12% of total isolates) was more extended than erythromycin resistance (8.7%) among the present isolates. In total, eighteen *emm* types were associated with tetracycline resistance (*p* ≤ 0.05), and nine were with erythromycin resistance [13]. As has been commonly described [12,13], *tet*M was more prevalent than *tet*O (185 vs. 81 isolates). No significant differences were seen among the MICs of the isolates with the *tet*M, *tet*O, or *tet*M-*tet*O genotypes (Table 1), suggesting that *tet*M and *tet*O provide similar resistance but have no additive or synergistic effects. Tetracycline and erythromycin co-resistance can be explained by the presence of MGEs that carry the *tet*M–*erm*B and *tet*O–*erm*TR genetic associations [6,7,8,9,14] (represented by *emm*11 and *emm*77, respectively).

The main resistant clones detected in this work (Table 2) have been described as being globally distributed [26]. Their temporal fluctuation in terms of prevalence conditioned the resistance rates recorded over the 14-year study period [11,13,24] (Figure 3 and Figure 4). Over this period, the highest tetracycline, erythromycin, and clindamycin resistance rates were, for the most part, owed to the presence of the *emm*11/ST403/*tet*M-*erm*B/MLSBc clone [10], the most representative co-resistant clone in numerical terms. The strong presence of the *emm*4/ST39/*mef*A-*msr*D/M, *emm*12/ST36/*mef*A-*msr*D/M, and *emm*28/ST52/*erm*B/MLSBc clones explains the high rates of erythromycin resistance seen during the first 4-year period. *emm*4 and *emm*12 conditioned the predominance of the M phenotype in 2007–2008, while *emm*11 and *emm*28 conditioned that of cMLSB in 2009–2010 (Figure 2, Figure 3 and Figure 4). In general, resistance rates declined in 2011–2015 and 2016–2020, although some exceptions were registered. In 2011, the highest tetracycline resistance rate was detected (21.8%, Figure 3) owing to the over-representation of the *emm*77/ST63/*tet*O clone (Figure 4), which was involved in two geographically distant surgical outbreaks. This majority clone (55% of *emm*77 isolates, Table 2), which was only resistant to tetracycline, was detected in 2007–2013. However, since 2013, *emm*77/ST63/*tet*O has been replaced by the *emm*77/ST63/*tet*O-*erm*TR/iMLSB and *emm*77/ST63/*tet*M-*tet*O-*erm*TR/iMLSB co-resistant clones, which have acquired the *erm*TR gene. This explains the slight increase in erythromycin resistance (iMLSB phenotype) detected during 2016–2020. Finally, the small, constant number of isolates of the *emm*22/ST46/*tet*M clone seemed to have little effect on tetracycline resistance in the period of 2007–2020.

The present study suffers from the limitation that MLST was only performed on a limited number of isolates; it is likely that the typing of all resistant isolates would have revealed wider genetic diversity. Furthermore, while this work provides a general view of antimicrobial resistance and its associated genes in Spain, the results shed no light on the MGEs involved in the transmission of these genes, which remain unknown [6,7,8,9]. Whole-genome sequencing is needed if we are to better understand the genetic environment of the resistance genes. This is the first nationwide study on antimicrobial resistance in invasive *S. pyogenes* in Spain, and comparing it with similar studies has been difficult because some variables differed in important ways, i.e., geographical origin, invasive vs. non-invasive isolates, and study period. Additionally, the lack of knowledge on the treatment that patients received does not permit us to know its influence on susceptibility patterns.

## 4. Materials and Methods

### 4.1. Basic Microbiological Typing

Three hundred and fifteen resistant bacterial isolates (one isolate per patient and clinical episode) were analyzed by SPIGAS between 2007 and 2020 [18]. The M protein gene (*emm* type) was acquired by sequencing the 180 hypervariable nucleotides of the gene’s 5′ end by using the Center for Disease Control and Prevention’s protocol and database [27]. The exotoxin genes, *spe*A, *spe*C, *spe*G, *spe*H, *spe*J, *sme*Z, and *ssa,* were detected by PCR [19,28]. A basic antibiogram was selected according to clinical and epidemiological criteria. Penicillin G, tetracycline, erythromycin, and clindamycin susceptibilities were tested using E-test strips (bioMérieux, Marcy-l’Etoile, France) following the recommendations and interpretative criteria of the European Committee on Antimicrobial Susceptibility Testing (EUCAST) [20]. Penicillin G, erythromycin, and clindamycin were mainly selected according to clinical—i.e., treatment—criteria, and tetracycline was selected according to epidemiological criteria.

### 4.2. Tetracycline, Erythromycin, and Clindamycin Resistance: Phenotype and Genotype Analyses

Isolates resistant to tetracycline (MIC > 1 mg/L) were checked by PCR for the presence of *tet*M [29] and *tet*O [30]. Isolates resistant to erythromycin (MIC > 0.5 mg/L) were analyzed to determine their phenotypes with respect to resistance to macrolides, lincosamides, and B streptogramins (M, cMLSB, and iMLSB phenotypes) by using the erythromycin–clindamycin double-disk test [25]. They were also examined by PCR for the presence of *msr*D [31], *mef*A [30], *erm*B [32], *erm*TR [16], and *erm*T [32].

### 4.3. Multilocus Sequence Typing and Description of Resistant Clones

Multilocus sequence typing (MLST) [33] included the amplification and partial sequencing of the seven housekeeping genes *gki*, *gtr*, *mur*I, *mut*S, *rec*P, *xpt*, and *yqi*L [34]; each allelic combination corresponded to a specific sequence type (ST). An MLST analysis was performed on a number of isolates of the majority resistant *emm* types, i.e., nine *emm*4-, seven *emm*11-, five *emm*12-, four *emm*22-, four *emm*28-, and seven *emm*77-type isolates. The resistant clones were identified by the combination of their *emm* type, ST, tetracycline- as well as macrolide-resistant genotype, and macrolide-resistant phenotype. It was assumed that isolates that shared their *emm* type, genotype, and phenotype would have identical STs.

### 4.4. Statistical Analysis

Categorical variables (*emm* type, age, gender, clinical manifestation, clinical sample, MICs, ARGs, and macrolide-resistance phenotype) were compared using the Fischer exact test. Age was grouped into six ranges: 0–4, 5–14, 15–39, 40–64, 65–74, and ≥ 75 years old. Significance was set up at *p* ≤ 0.05. All calculations were carried out by using Stata v.17 software.

### 4.5. Ethical Approval

Bacterial strains were collected as part of standard patient care and were sent to a public national reference laboratory (Centro Nacional de Microbiología, Majadahonda, Spain) for microbiological typing. This study focused on bacteria, and no identifiable human data were used; ethical approval was, therefore, not required.

## 5. Conclusions

Over the study period, erythromycin resistance in invasive *S. pyogenes* in Spain was found to be clustered in the clones with the *emm*4, *emm*11, *emm*12, and *emm*77 genotypes; the genes most commonly involved (in descending order) were *erm*B, *mef*A, and *erm*TR. Macrolide-resistant cMLSB was the most frequently detected phenotype. Clindamycin resistance was always mediated by methylases. *emm*11, *emm*22, and *emm*77 were associated with tetracycline resistance, with the *tet*M gene more extended than *tet*O. Tetracycline–erythromycin resistance was common and grouped in the clones with the *emm*11 and *emm*77 genotypes. Tetracycline, erythromycin, and clindamycin resistance rates declined from 2007 to 2020, with temporal variations in resistant clones corresponding to changes in resistance rates.

## Figures and Tables

**Table 1:** *emm* types and genotypes of invasive *Streptococcus pyogenes* associated with tetracycline, erythromycin, and clindamycin resistance in Spain, 2007–2020.

Type ^a^	N ^b^	Tetracycline ^c^				Erythromycin ^c^				Clindamycin ^c^			
		*n*	%	MIC_50_	MIC_90_	*n*	%	MIC_50_	MIC_90_	*n*	%	MIC_50_	MIC_90_
***emm***													
Total	1983	237	12.0	0.25	12	172	8.7	0.125	0.25	78	3.9	0.125	0.25
*emm*4	161	1	0.6	0.25	0.5	24	14.9	0.125	16	0	0.0	0.125	0.25
*emm*11	63	51	81.0	16	32	47	74.6	>256	>256	45	71.4	>256	>256
*emm*12	115	1	0.86	0.25	0.5	18	15.7	0.19	16	2	1.7	0.125	0.25
*emm*22	36	11	30.6	0.25	16	2	5.6	0.125	0.25	1	2.8	0.125	0.25
*emm*28	97	2	2.1	0.25	0.5	11	11.3	0.125	>256	9	9.2	0.125	0.5
*emm*77	81	74	91.4	32	32	23	28.4	0.19	16	1	1.2	0.125	0.25
**Genotype**													
*tet*M	-	152	-	16	48	-	-	-	-	-	-	-	-
*tet*O	-	48	-	32	48	-	-	-	-	-	-	-	-
*tet*M-*tet*O	-	33	-	24	32	-	-	-	-	-	-	-	-
*mef*A-*msr*D	-	-	-	-	-	40	-	16	32	0	0	0.125	0.25
*erm*B	-	-	-	-	-	61	-	>256	>256	57	-	>256	>256
*erm*TR	-	-	-	-	-	24	-	12	>256	1	-	0.125	0.25
*erm*T	-	-	-	-	-	1	-	>256	>256	0	0	0.125	0.125

MIC, minimum inhibitory concentration; S, susceptible; R, resistant; and -, not analyzed. ^a^: Data are for the majority *emm* types significantly associated (*p* ≤ 0.05) with tetracycline, erythromycin, and/or clindamycin resistance, as well as for the most representative genotypes. ^b^: Total number of isolates. ^c^: The number (*n*) and percentage (%) of resistant isolates are indicated for each antibiotic. MICs are expressed in mg/L. Antimicrobial susceptibility is interpreted according to EUCAST criteria [20]. Tetracycline: ≤1, S; >2, R. Erythromycin: ≤0.25, S; >0.5, R. Clindamycin: ≤0.5, S; >0.5, R.

**Table 2:** Data of the main resistant clones of invasive *Streptococcus pyogenes* in Spain, 2007–2020.

*emm* Type	No. Isolates ^a^	MLST ^b^		Genotype ^c^							Phenotype ^d^
		ST	*n*	*tet*M	*tet*O	*msr*D	*mef*A	*erm*B	*erm*TR	*erm*T	
Only tetracycline resistance											
*emm*22	10/12	46	2	+	-						
*emm*77	41/74	63	3	-	+						
Only erythromycin resistance											
*emm*4	19/25	39	5			+	+	-	-	-	M
*emm*12	12/18	36	4			+	+	-	-	-	M
*emm*28	6/11	52	2			-	-	+	-	-	cMLSB
Tetracycline–erythromycin co-resistance											
*emm*11	33/51	403	5	+	-	-	-	+	-	-	cMLSB
*emm*77	8/74	63	2	-	+	-	-	-	+	-	iMLSB
*emm*77	8/74	63	2	+	+	-	-	-	+	-	iMLSB

^a^: Assumed number of clonal isolates with respect to the total number of resistant isolates. ^b^: MLST, multilocus sequence typing; ST, sequence type; and *n*, number of isolates analyzed by MLST for each clone. ^c^: +, present; -, absent. ^d^: Macrolide-resistant phenotype. M, M phenotype; cMLSB, constitutive MLSB phenotype; and iMLSB, inducible MLSB phenotype.

## Data Availability

Not applicable.
